# Minimizing H.R.1-related Medicaid coverage disruptions for high-risk patients

**DOI:** 10.1093/haschl/qxag162

**Published:** 2026-07-01

**Authors:** Rachel M Everhart, Sarah Gillespie, Marcella Maguire, Sarah A Stella, Rebecca Hanratty, Tracy L Johnson

**Affiliations:** Ambulatory Care Services, Denver Health, Denver, CO 80204, United States; Department of Medicine, University of Colorado Anschutz Medical Campus School of Medicine, Aurora, CO 80045, United States; Housing and Communities Division, Urban Institute, Washington, DC 20024, United States; Strategy and External Affairs, Corporation for Supportive Housing, NewYork, NY 10006, United States; Department of Medicine, University of Colorado Anschutz Medical Campus School of Medicine, Aurora, CO 80045, United States; Department of Medicine, Denver Health, Denver, CO 80204, United States; Ambulatory Care Services, Denver Health, Denver, CO 80204, United States; Department of Medicine, University of Colorado Anschutz Medical Campus School of Medicine, Aurora, CO 80045, United States; Department of Medicine, University of Colorado Anschutz Medical Campus School of Medicine, Aurora, CO 80045, United States; Health Policy Solutions, Golden, CO 80403, United States

**Keywords:** Medicaid coverage, work requirements, homeless, enrollment support, cross-sector coordination, data integration

## Abstract

Forthcoming H.R.1 Medicaid eligibility changes requiring documentation of work, school, or volunteer activities are projected to result in significant disenrollment. High-risk beneficiaries, including those experiencing homelessness, are more likely to experience barriers in navigating the eligibility process, such as qualifying for work requirement exemptions. We utilized an existing set of linked data across the medical safety net, state Medicaid agency, housing services providers, and justice agencies as a case study to understand the prevalence of select potential medical and social exemptions from work requirements, examine safety net system utilization to identify potential touch points to prevent coverage loss, and estimate medical costs to inform planning and resourcing efforts to support continued enrollment. We found a high prevalence of Medicaid members potentially exempt from work requirements and of interactions with the medical safety net and with housing service providers specifically within the group experiencing homelessness. Increased medical costs that would likely shift to the safety net if coverage loss occurred demonstrate an opportunity for community efforts to support enrollment. Our case study can serve as a model for other communities proactively to plan for the local impact of H.R.1 eligibility changes, including data integration, coordination, and support efforts.

## Teaser

Health and housing service providers can plan and coordinate to minimize H.R.1-related coverage loss for high-risk Medicaid beneficiaries.

Key Points

- Communities can begin planning and implementing coordinated efforts across health and housing service providers to minimize H.R.1-related coverage loss for high-risk Medicaid beneficiaries.
- Potential exemptions from Medicaid eligibility work requirements are prevalent and safety net providers can support communication and documentation.
- Cross-sector data integration and coordination will facilitate consolidating outreach and enrollment support efforts that are likely to be resource constrained.

## Introduction

In July 2025, US Congress passed H.R.1,^1^ introducing major Medicaid eligibility changes including community engagement provisions that require documentation of work, school, or volunteer activities (hereafter, “work requirements”) for adults who gained eligibility under the Affordable Care Act based on incomes up to 138% of the federal poverty level ($22 024.80 for a family size of 1^2^). H.R.1 also specifies criteria excluding individuals from work requirements including caretaker status, medical frailty, incarceration, and pregnancy. On June 1, 2026, an interim final rule with comment period (IFC) was released interpreting the H.R.1 statute related to work requirements imposing considerations of severity and functional capacity to the definition of the medically frail exemption.^3^ The Congressional Budget Office projects that more than 5.2 million people will lose Medicaid coverage over the next decade due to inability to meet or prove exemption from work requirements, many of whom will become uninsured.^4^ To reduce the disenrollment risk among eligible members, H.R.1 directs state Medicaid agencies to use automated systems to screen for compliance with these provisions or exemptions and to proactively outreach enrollees, ideally coordinating with local service providers, before rolling out work requirements.

Potential exemptions from work requirement documentation are relevant to the broader Medicaid beneficiary population and particularly to the subset experiencing homelessness. H.R.1 does not include homelessness in the criteria excluding individuals from work requirements. The IFC explicitly states that the experience of homelessness is not a medical condition and cannot deem a person as medically frail, but individuals experiencing homelessness may have a medically frail condition qualifying them for the exclusion. Past research on work requirements in other public programs reveals that this population is disproportionately disenrolled because their lack of stable housing complicates their ability to prove their identity, address, citizenship, work, or exemption status.^5,6^ Loss of Medicaid coverage for many individuals experiencing homelessness would not only result in losing access to medical care but also housing-related services, given that many states have approval for Medicaid to cover these as a strategy to improve health outcomes and control costs.^7,8^

The health and housing sectors can play crucial support roles throughout the Medicaid eligibility process by identifying those at risk of coverage loss, making the business case to plan and budget outreach resources, and conducting enrollment support to high-risk individuals. Previous research demonstrates that automated methods, paired with systematic, data-informed coordination across clinical, social service, and community partners, improve Medicaid retention at renewal.^9^ This requires more robust cross-sector data-sharing and collaboration than typically exists in many communities but is feasible^10^ and necessary to prevent coverage loss.

Health and homeless service providers should not wait until federal and state guidance is finalized to begin planning and preparing to support their clients. However, they will need to understand operational implementations and have access to relevant enrollment and eligibility information. Linked health and housing data may increase communities' ability to identify people experiencing homelessness, identify Medicaid enrollment status, verify exemption and compliance status, and minimize disruption to enrollment and services.

To understand what proactive preparation could look like in practice, we leveraged an existing linked administrative dataset as a descriptive case study. In light of the case study analysis and findings, we discuss how communities can prepare to minimize disruption in Medicaid coverage.

## Methods

Our case study utilized a historical 2021 dataset created as part of a prior project focused on data analytics to support the alignment of high-risk adult programs and services. This work was centered at Denver Health, a large urban safety-net health system serving a high proportion of Medicaid members, including those experiencing homelessness. Specifically, we leveraged data-sharing agreements and matched data between Denver Health's electronic health records (EHRs), the state Medicaid office (Department of Health Care Policy and Financing), the regional Homeless Management Information System (HMIS), and criminal justice agencies (state Department of Corrections, municipal jail system).

In the current analysis, the adult Medicaid Expansion group, ages 19 to 64 years, subject to future work requirements was identified based on the presence of modified adjusted gross income (MAGI) adult as the primary eligibility description available via monthly rosters of fee-for-service Medicaid members attributed primarily through historical claims to Denver Health in 2021 as a primary care medical provider (PCMP) under the state's accountable care collaborative.^11^ MAGI adult eligibility was deemed to be a valid estimation of the Medicaid Expansion group in the historical dataset based on data supplied in the current monthly roster where the Medicaid Expansion group is newly flagged; 97.5% of the MAGI adult eligibility group is flagged as Medicaid Expansion and 96.8% of the Medicaid Expansion group has MAGI adult eligibility.

We modeled a subset of H.R.1-identified medical and social factors that could potentially exempt members from work requirements. Individuals could be identified in multiple groups, specifically the following:

- Medically frail: based on EHR diagnoses of mental health (MH) or substance use disorders (SUDs) identified through *International Classification of Diseases, Tenth Revision* (ICD-10) codes, as an encounter diagnosis in the past 3 years or a problem list diagnosis active as of the historical 2021 time frame. Mental health was identified based on ICD-10 codes F20–F99 or R45–R46. Substance use disorder was identified based on ICD-10 codes F10–F19, excluding F17 codes for nicotine dependence. Medically frail was also identified through the 3M clinical risk group (CRG) severity-adjusted diagnosis grouper to approximate individuals with serious or complex medical conditions.^12^ Denver Health uses the CRG to assess health risk for delivery system administrative purposes, including determining primary care practice panel sizes and risk, segmenting the population for care management services. Prior research has validated that people identified as medically complex in this diagnosis grouper manner have increased risk of hospitalization and total costs.^13^ The CRG was analyzed as the grouper's core health status group concept. These groups range from 1 to 9 corresponding to increasing number of medical conditions and severity. We limited to groups of 6–9, reflecting significant or dominant chronic disease in multiple organ systems, dominant and metastatic malignancies, and catastrophic conditions.
- Recent incarceration: identified via an EHR corrections registry populated based on release in the past 12 months per justice agency release files. This registry was developed to support a corrections transition clinic and model a continuum of care after release.^14^ Although the work requirement exemption is specific to current incarceration or within 3 months, this was the scope of the data in our historical dataset and used as a proxy.
- Pregnant women: based on EHR documentation of delivery in the prior 12 months or an EHR obstetrical episode active during the 2021 historical time frame. Evidence of pregnancy in the EHR was selected given that this exemption status could be missed by state algorithms which may rely on pregnancy-specific eligibility codes and miss pregnant people who qualified through Medicaid Expansion specific eligibility codes.
- Caregivers: adults with the same Medicaid household identifier as a child aged 13 years or younger with at least a 15-year age difference between the adult and any children within the same Medicaid household. The age difference was included to identify parents/caregivers as opposed to older siblings living in the same household. We did not model other exempt caregiver groups, such as caregivers of older children or adults with disabilities.
- Medicare enrollees: based on the flag indicating Medicare enrollment in a monthly Medicaid roster or EHR encounter in 2021 with Medicare financial class.

To understand the particular risks for people experiencing homelessness, we also identified this population. Homelessness was determined through HMIS documentation of services for shelter, street outreach, or housing (including permanent supportive housing, rapid re-housing, other permanent housing, transitional housing, and safe haven) or through a robust EHR-based registry inclusive of registration-based information, geocoded matching of patient addresses to housing services providers, homeless diagnosis (Z59.0X), nursing or care management assessments, and health-related social needs screening.^15^ Any evidence of homelessness during the 2021 historical time frame was used for identification. Although this method likely undercounts people experiencing homelessness, it includes a broad representation across the medical safety net including assessments and screenings taking place within the emergency department and the social safety net given the centralization of data within HMIS for all recipients of federal Housing and Urban Development (HUD) dollars under the Continuum of Care (CoC) Program.

We calculated the prevalence of the select potential work requirement exemptions within the broader adult Medicaid Expansion population as well as within the subset of this population experiencing homelessness. To inform potential health system touchpoints, we calculated the prevalence of key health care utilization, including primary care, emergency department, or inpatient admission. Utilization data were sourced from the Denver Health EHRs and supplemented with Medicaid data for the emergency department and hospital admission outcomes. We also calculated the prevalence of utilization of homeless services via HMIS documentation inclusive of not only the service providers detailed previously to identify homelessness (shelter, street outreach, and housing) but also HMIS-documented services for homelessness prevention or standalone supportive services, such as childcare, employment assistance, or transportation. To estimate the potential financial impact of coverage loss, we leveraged data that had been shared by the state Medicaid agency to support the historical project. We calculated Medicaid total cost of care in the historical 2021 time frame from the payer perspective inclusive of all utilization and costs and summarized as medians and interquartile ranges (IQRs) per member per month (PMPM) based on months of Medicaid attribution in 2021 for the different groups. This case study was embedded within a broader project, which was reviewed and approved by the Colorado Multiple Institution Review Board.

Our case study has several limitations. We only modeled select work requirement exemption categories and did so with the data available to us through a prior project and based on the lens of where the safety net may be able to inform Medicaid eligibility with data the state may not have access to. Our methods may have mischaracterized some of the categories. We did not account for the functional capacity consideration of medically frail as imposed by the IFC. Our methods also may have incorrectly identified homelessness given the transient nature of that experience and our awareness of it only through specific point-in-time contacts with the safety net. Although our analysis includes several external data sources, the EHR data are from a single institution and do not reflect diagnoses, assessments, or utilization from other health care delivery sites. Conversely, our case study is based on a group of Medicaid enrollees who are attributed to the delivery system and that attribution is largely based on prior claims with the institution. Therefore, our estimates of utilization and connection to the system which can help inform potential reach may be overstated for a broader Medicaid enrollee population that may not have engaged in care. Finally, the existing data that we leveraged were from 2021, while Medicaid continuous coverage was in effect in response to the COVID-19 pandemic.

## Results

**Figure 1.**
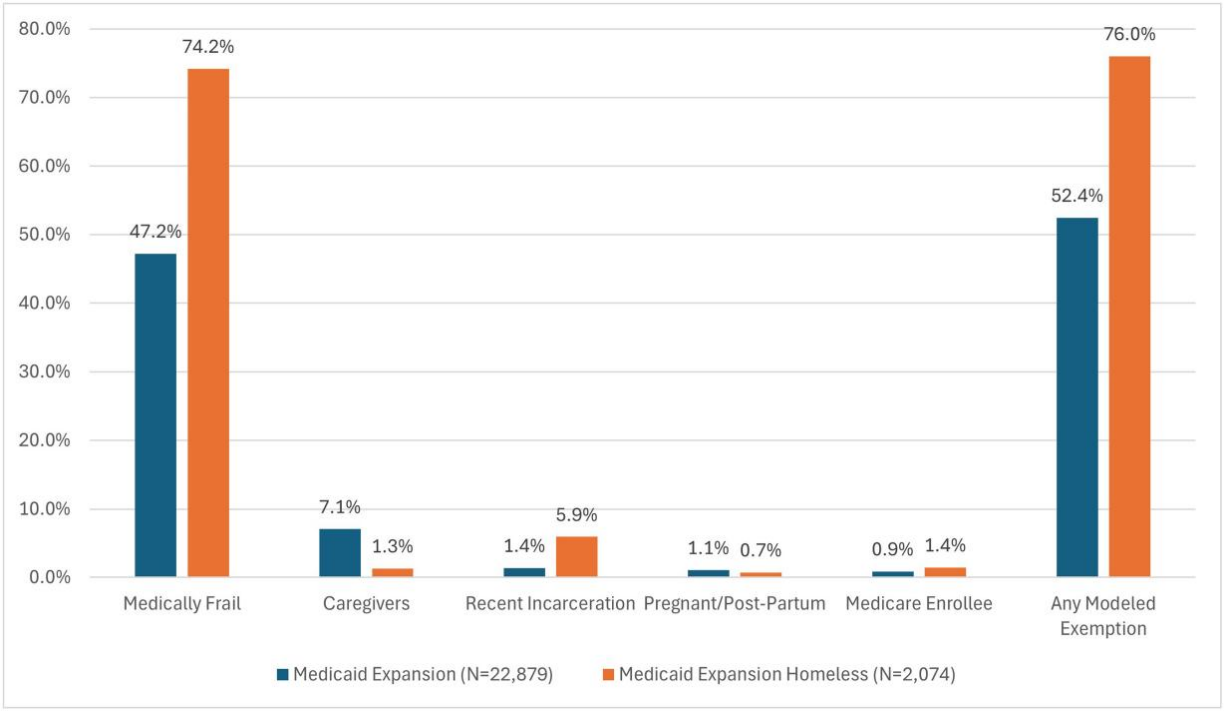
Select H.R.1-identified medical and social factors that potentially exempt members from meeting work requirements among Denver Health 2021 Medicaid-attributed enrollees (*n* = 71 727) eligible through expansion criteria and within the subpopulation experiencing homelessness.

Of 71 127 Colorado Medicaid members who were attributed to Denver Health in 2021, nearly one-third (32.2%) were enrolled under the Medicaid Expansion group, ages 19 to 64 years, that must document work or exemption status. The steps to identify the population are summarized in Figure S1. Of the 22 879 individuals in the Medicaid Expansion group, 52.4% have at least 1 of the select medical and social factors modeled that may indicate work requirement exemption (Figure 1). Nearly half (47.2%) were classified as potentially medically frail (with catastrophic, cancer, multiple chronic health, or SUD/MH conditions) and 7.1% as caregivers. Recent incarceration status (1.4%), pregnancy (1.1%), and dual Medicare enrollment (0.9%) were much less prevalent. Among people experiencing homelessness (*n* = 2074), three-quarters (76.0%) were classified as meeting any modeled potential work requirement exemption. Sociodemographic characteristics of the cohorts are summarized in Table S1.

**Figure 2.**
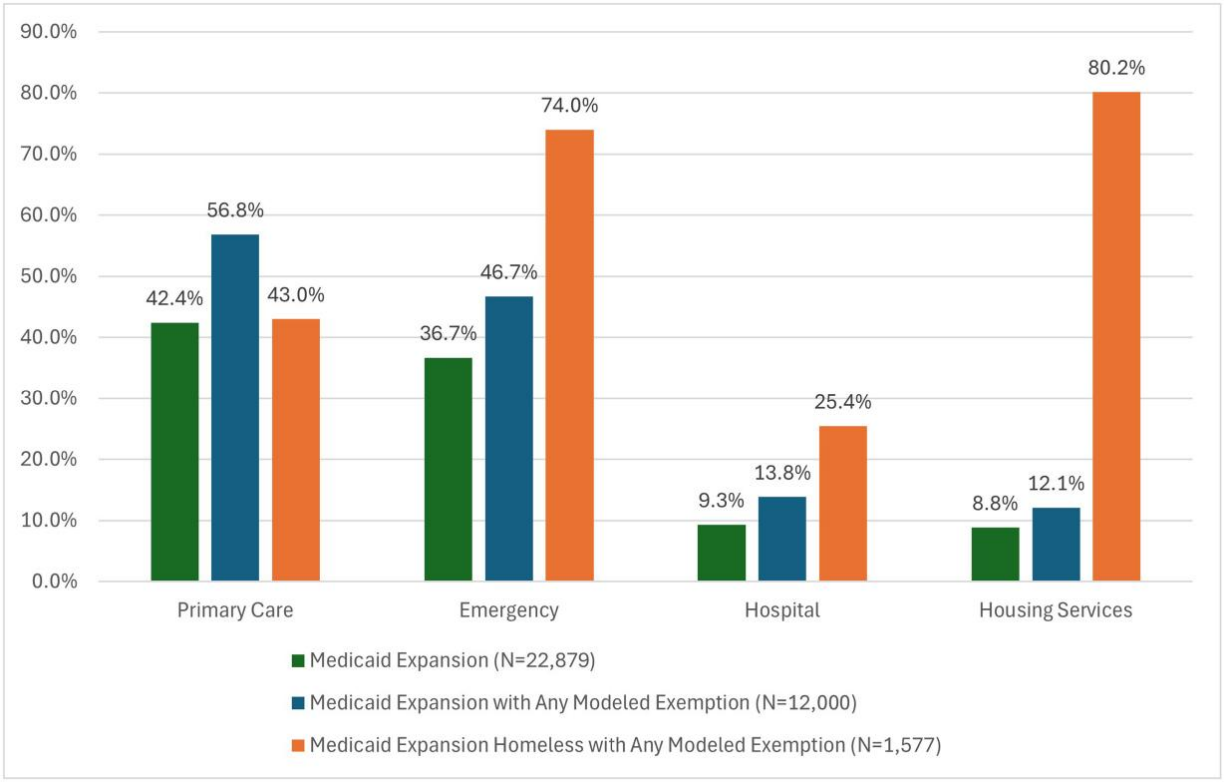
Health care and housing services utilization among Denver Health 2021 Medicaid attributed enrollees (*n* = 71 127) eligible through expansion criteria and within the subpopulations (1) modeled with select H.R.1-identified medical and social factors that exempt members from meeting work requirements and (2) within those experiencing homelessness.

Among the Medicaid Expansion population with any modeled exemption, primary care utilization was most prevalent (56.8%) followed by emergency department utilization (46.7%) and hospital admission (13.8%) (Figure 2). This pattern was similar for the broader Medicaid Expansion population, but they utilized less services across all service categories. Among the cohort experiencing homelessness, emergency utilization was highly prevalent (74.0%), primary care utilization was less prevalent (43.0%), and hospital admission was more prevalent (25.4%) than within the broader population. Utilization of housing services, including shelter, street outreach, housing, prevention, and supportive services, was also highly prevalent (80.2%) within the subset experiencing homelessness.

Among Medicaid Expansion enrollees, members with any modeled exemption had a median total cost of care of $302.43 PMPM (IQR: $165.99–$671.02) and $468.35 PMPM (IQR: $230.12–$1182.19) for those experiencing homelessness, compared with a population median of $207.40 PMPM (IQR: $118.25–$435.84).

## Practical implications

Our case study illustrates the safety net's high prevalence of Medicaid enrollees who are at risk of coverage disruption or loss due to new administrative processes required by H.R.1 and who are potentially exempt from future work requirement documentation. The utilization review highlighted frequent interaction with the health system and also with housing service providers among the homeless cohort. The analysis of total cost of care demonstrated higher costs in the group who may qualify for an exemption and was further pronounced within the subpopulation experiencing homelessness. These findings can serve as a model for other health and homeless service providers to consider how to prepare to support their communities to minimize coverage disruptions.

### Leverage existing data to estimate who is most vulnerable to coverage loss

This analysis demonstrates how communities can approach identifying patients at high risk of losing Medicaid coverage. This serves not only to flag likely exemptions but also to identify potential system solutions and to focus outreach efforts. Of the select medical and social exemptions we modeled, medical frailty was the most prevalent. These people are particularly at risk of adverse health outcomes if they lose Medicaid coverage under H.R.1. Some of this “high-risk” group may need health system support in documenting medical conditions for work requirement exemption. Alternatively, nonexempt, high-risk patients may need assistance navigating new work requirement documentation procedures to prevent harmful coverage gaps.

### Make the business case for enrollment support

Knowing who is at risk of coverage loss is only the first step: health systems can also make the business case for institutional and community investment in enrollment support, especially since federal funding for eligibility assistance has been cut.^16^ Our examination of utilization and cost experience suggests that Medicaid coverage loss would be medically and financially detrimental for patients who may be exempt from work requirements and for the safety-net health systems they rely on. Total cost of care was higher in the cohort modeled with any exemption compared with the broader Medicaid Expansion population, indicating that more health care services are utilized. This effect is more pronounced in the population of members experiencing homelessness; these members incur health spending more than 100% greater than the broader Medicaid Expansion population. Coverage disruptions could lead to reduced access to ongoing treatment, increased medical debt, and higher and unsustainable uncompensated care burdens for the medical safety net.

Beyond the financial case, our findings point to concrete, underutilized channels for reaching vulnerable patients before coverage disruptions occur. In particular, high rates of emergency department use and homelessness services engagement among high-risk homeless patients indicate frequent interaction with and potential opportunity to leverage existing partnerships between health and housing systems. These points of contact represent strategic opportunities to provide Medicaid enrollment support, particularly if staff are equipped with information, tools, and resources to meaningfully assist. Utilization of primary care is also most prevalent for the broader population that may qualify for an exemption and that setting may facilitate more appropriate eligibility support than can be provided in an acute care setting.

### Integrate data to facilitate shared efforts

Traditionally siloed information about Medicaid beneficiaries can be shared and integrated across payer, health, and social platforms to aid in focusing and coordinating outreach efforts and documentation needs related to eligibility and enrollment, but these efforts typically require cross-organization planning and implementation.

Data sharing between Health Insurance Portability and Accountability Act (HIPAA)–covered health systems and community partners providing care coordination to address health-related social needs has been difficult to navigate given that HIPAA Privacy Rule–proposed changes published in January 2021 are not yet finalized.^17^ Communities with a robust community or social health information exchange may be able to leverage this resource for data sharing and integration, but these systems face implementation barriers and are not yet broadly available.^18^ Alternatively, health systems may elect to put in place other safeguards related to data sharing with community care coordination partners, such as updating their Notice of Privacy Practices to expressly communicate care coordination data sharing, identifying key community partners to execute appropriate legal agreements such as business associate or data use agreements, and/or implementing workflows to collect patient-level release of information documentation.

To optimize outreach and support offered to Medicaid beneficiaries, health system and housing service providers will need states and health plans to share eligibility information every month. These data should include work requirement eligibility, Medicaid renewal dates, and detailed state criteria to define and document exemptions such as medical frailty. If states implement these processes primarily through manual workflows, health systems and providers would greatly benefit from additional funding to manage the volume, particularly during start-up. Providers can also begin planning for how this information can be ingested and/or made visible to staff to support the intended workflows. For example, health system registration staff who focus on scheduling and checking-in a patient for a visit may be the best resource for communicating eligibility reminders to patients, but they likely do not have visibility or regularly access the same screens and tools in the EHRs as clinical staff.

### Identify health system outreach opportunities to prevent coverage loss

Given that there is unlikely to be substantial new funding for health systems to provide Medicaid enrollment support, building on existing workflows will be essential. Many safety-net providers have developed operational processes and clinical workflows tailored to patients both at risk of adverse health outcomes and lack of coverage. In our illustrative setting, uninsured patients are already directed to enrollment support, primary care and hospital staff can access information about patient risk and homelessness status, case managers receive referrals based on various risk assessments, patients are given personalized visit summaries with follow-up instructions plus recommended medical and community services, and automated outreach campaigns send patient portal and text message reminders of to-do actions. Safety-net providers such as hospital staff who engage in Medicaid presumptive eligibility activities are likely to have an additional role in supporting patients in navigating work requirements and potential exemptions. These and other workflows could be further adapted to more effectively assist patients who need help applying for or renewing Medicaid, including focused outreach to high-risk populations.

Furthermore, health systems are especially well positioned to provide documentation for certain exemptions—for example, due to medical frailty or pregnancy. Health systems can begin developing interdisciplinary workflows and templated attestations based on EHR diagnoses, disease severity, and functional capacity. Existing workflows for templated clinical documentation, such as well child visits, and previsit questionnaires, such as those used for Medicare annual wellness visits, could be modified to support required documentation. Although state Medicaid agencies are likely to have some visibility into this EHR information via covered claims, diagnoses may predate coverage or this documentation may be required until more automated processes such as claims-based algorithms are developed. Planning can begin to make these resources available via patient portals and the EHR to all members of the care team so that patients do not have to repeatedly schedule an appointment with their provider to secure this documentation that may be needed every 6 months for re-enrollment.

### Identify housing service provider outreach opportunities to prevent coverage loss

Housing service providers have additional opportunities to offer enrollment-support opportunities that health systems cannot fully replicate. But they too will need critical information, guidance, and support to assist.

Some shelters and supportive housing programs assist clients in applying for benefits and therefore understand the Medicaid enrollment process. Because some also collect and distribute mail on behalf of residents, they are well positioned to flag Medicaid enrollment packets and required documentation requests before deadlines pass. Staff at these programs often have established trusted relationships with residents who may be less likely to engage with clinical settings, making them effective first-line navigators. Some homeless services organizations are creating opportunities for work/community engagement and may be able to support documenting hours for the population who will not be exempt from work requirements. Programs that already employ case managers or benefit navigators could formalize an application assister role, while those without existing capacity could partner with Medicaid enrollment sites, legal aid organizations, or other community partners to provide assistance. Training for these trusted messengers will need to be developed and offered in a language that is understandable to this audience. Furthermore, an individual receiving services from both health and housing partners may benefit from coordinated coverage-related outreach and support and the systems may benefit from shared, unduplicated efforts. The health system that our case study was embedded within has experience coordinating with housing partners through data integration, identification of shared clients at the point of care, documentation of release of information, and an immediate in-person connection to a housing partner.^8^

## Conclusion and considerations for states

Prior research on state waiver programs has warned that work requirements are likely to disenroll the very people who may qualify for exemption and are less able to navigate new administrative requirements.^19,20^ This includes the medically frail population that our case study identified as prevalent within the safety net.

While H.R.1 directs state Medicaid agencies to leverage automated “ex parte” eligibility processes—including screening for exemptions—states face complex and costly systems changes, with few staff and tight timelines.^21^ For example, best practices for identifying exempt populations and addressing data lags may require linking to external data sources.^22^ Some data are not likely to be available electronically, at least in the short term, such as functional capacity. As a result, most states plan to launch a “minimum viable product” to meet the legislative deadline of January 2027, with subsequent refinements over time. In the short term this will likely mean collecting compliance and exemption information manually—for example, through “auditable self-declaration”—until more seamless data exchanges can be established.

Housing and homelessness sectors can potentially serve as first-line navigators for their affected clients. Health care providers offer a complementary line of defense and can provide helpful information for shared clients. Both sectors will need training, data tools, and ideally, financial support to be effective. Training must be in plain language that connects insurance coverage with access to health and supportive services. Tools should be easily integrated into existing data and documentation systems. Collaborations between the health and housing sectors are both possible and well poised to create the partnerships needed to maintain coverage.

We demonstrate that assessing potential exemption status with linked data, including data sources from Medicaid, health care, regional HMISs, and justice agencies, is both feasible and essential to prevent harmful and costly coverage gaps, particularly for individuals who are likely to be exempt. Populations that are experiencing homelessness may especially benefit when states adopt robust ex parte processes that include automated exemption screening as well as work verification. To mitigate disruption in services, states and communities must collaborate to identify people experiencing homelessness and other high-risk populations, strengthen ex parte processes, and implement cross-sector outreach for exemption/compliance processes.

## Supplementary Material

qxag162_Supplementary_Data

## Data Availability

The de-identified dataset is available upon request.

## References

[1] US Congress . H.R.1—119th Congress (2025-2026). An Act to provide for reconciliation pursuant to title II of H. Con. Res. 14. Accessed April 7, 2026. https://www.congress.gov/bill/119^th^-congress/house-bill/1/text

[2] US Department of Health and Human Services, Office of the Assistant Secretary for Planning and Evaluation . 2026 Poverty guidelines: 48 contiguous states (all states except Alaska and Hawaii). Accessed June 1, 2026. https://aspe.hhs.gov/sites/default/files/documents/b1bfa16b20ae9b89d525bc35de7c1643/detailed-guidelines-2026.pdf

[3] Department of Health and Human Services, Centers for Medicare & Medicaid Services . Medicaid program; community engagement requirement for certain individuals. Accessed June 3, 2026. https://www.federalregister.gov/documents/2026/06/03/2026-11094/medicaid-program-community-engagement-requirement-for-certain-individuals

[4] Congressional Budget Office . Estimated effects on the number of uninsured people in 2034 resulting from policies incorporated within CBO's baseline projections and H.R. 1, the One Big Beautiful Bill Act. June 4 2025. Accessed October 13, 2025. https://www.cbo.gov/publication/61463

[5] Gray C, Leive A, Prager E, Pukelis K, Zaki M. Employed in a SNAP? The impact of work requirements on program participation and labor supply. Am Econ J Econ Policy. 2023;15(1):306–341. 10.1257/pol.20200561

[6] Maguire M, Barkoff A. Medicaid's new address verification requirements could impose significant burdens. Health Aff Forefront. 2025. 10.1377/forefront.20251001.497575.

[7] Charania S . How Medicaid and states could better meet health needs of persons experiencing homelessness. AMA J Ethics. 2021;23(11):E875–E880. 10.1001/amajethics.2021.875 34874257

[8] Corporation for Supportive Housing . Summary of state actions: Medicaid & housing services. Updated spring 2025. Accessed April 23, 2026. https://insights.csh.org/hubfs/Summit%20Resources%20to%20Share/CSH%20Summary%20of%20Medicaid%20State%20Actions%20-%20Spring%202025.pdf

[9] Ganguly A, Truchil A, Wiest D. Improving Medicaid renewal outreach: evidence from rapid-cycle testing in New Jersey. Camden Coalition. Updated March 2026. Accessed April 10, 2026. https://camdenhealth.org/resources/rapid-cycle-testing-medicaid-renewal/

[10] Gillespie S, Hanson D, Doñate A, Stella S, Gray T, Everhart R. Harnessing health care systems to enhance supportive housing. Urban Institute. Updated June 12, 2025. Accessed April 10, 2026. https://www.urban.org/research/publication/harnessing-health-care-systems-enhance-supportive-housing

[11] Colorado Department of Health Care Policy & Financing . Accountable care collaborative phase III. Accessed April 23, 2026. https://hcpf.colorado.gov/accphaseIII

[12] 3M Health Information Systems . 3M clinical risk groups: measuring risk, managing care. Updated September 2016. Accessed April 21, 2026. https://multimedia.3m.com/mws/media/1356109O/crg-measuring-risk-managing-care-ukv1.pdf

[13] Johnson TL, Brewer D, Estacio R, et al Augmenting predictive modeling tools with clinical insights for care coordination program design and implementation. EGEMS (Wash DC). 2015;3(1):1181. 10.13063/2327-9214.1181 26290884 PMC4537083

[14] Frank M, Loh R, Everhart R, Hurley H, Hanratty R. No health without access: using a retrospective cohort to model a care continuum for people released from prison at an urban, safety net health system. Health Justice. 2023;11(1):49. 10.1186/s40352-023-00248-3 37979038 PMC10656837

[15] Stella SA, Hanratty R, Davidson AJ, et al Improving identification of patients experiencing homelessness in the electronic health record: a curated registry approach. J Gen Intern Med. 2024;39(16):3113–3119. 10.1007/s11606-024-08909-1 39285073 PMC11618276

[16] Pestaina K . A 90% cut to the ACA navigator program. KFF. Quick takes: timely insights and analysis from KFF staff. Updated February 14, 2025. Accessed March 22, 2026. https://www.kff.org/quick-take/a-90-cut-to-the-aca-navigator-program/

[17] US Department of Health and Human Services . Proposed modifications to the HIPAA privacy rule to support, and remove barriers to, coordinated care and individual engagement. 86 FR 6446. p. 6446-6538. Accessed April 21, 2026. https://www.federalregister.gov/documents/2021/01/21/2020-27157/proposed-modifications-to-the-hipaa-privacy-rule-to-support-and-remove-barriers-to-coordinated-care

[18] Fichtenberg C, Delva J, Minyard K, Gottlieb LM. Health and human services integration: generating sustained health and equity improvements. Health Aff. 2020;39(4):567–573. 10.1377/hlthaff.2019.01594 32250685

[19] Hinton E, Rudowitz R. Implementing work requirements on a national scale: what we know from state waiver experience. KFF. Updated May 20, 2025. Accessed February 22, 2026. https://www.kff.org/medicaid/implementing-work-requirements-on-a-national-scale-what-we-know-from-state-waiver-experience/

[20] Musumeci M . Disability and technical issues were key barriers to meeting Arkansas' Medicaid work and reporting requirements in 2018. KFF. Updated June 11, 2019. Accessed February 22, 2026. https://www.kff.org/medicaid/disability-and-technical-issues-were-key-barriers-to-meeting-arkansas-medicaid-work-and-reporting-requirements-in-2018/#8dcda51e-9c1b-4fdb-a8ed-bc5708fbc8ef

[21] Diana A, Tolbert J, Hinton E, Rudowitz R. Challenges with implementing work requirements: findings from a survey of state Medicaid programs. KFF. Updated October 31, 2025. Accessed February 22, 2026. https://www.kff.org/medicaid/challenges-with-implementing-work-requirements-findings-from-a-survey-of-state-medicaid-programs/

[22] Serafi K, Frohlich JPB, McAvey KC, Sears L. How health information exchanges can identify medically frail work requirement exemptions. Manatt. Updated February 18, 2026. Accessed February 22, 2026. https://www.manatt.com/insights/newsletters/health-highlights/how-health-information-exchanges-can-identify-medically-frail-work-requirement-exemptions

