# Supplementary material for: Minimizing H.R.1-related Medicaid coverage disruptions for high-risk patients
Source: Health Aff Sch. 2026 Jul 1;4(7):qxag162. doi: 10.1093/haschl/qxag162 (PMC13367571; doi:10.1093/haschl/qxag162)
Supplement: qxag162_Supplementary_Data [file qxag162_supplementary_data.zip › Table_S1.docx]

Table S1. Sociodemographic characteristics of Denver Health 2021 Medicaid attributed enrollees eligible through Expansion criteria and the subpopulations experiencing homelessness and with select modeled H.R.1-identified factors that potentially exempt members from work requirements.

| Sociodemographic Characteristic | Medicaid Expansion, N (%) | | Medicaid Expansion Homeless, N (%) | |
| --- | --- | --- | --- | --- |
|  | All | Any Modeled Exemption | All | Any Modeled Exemption |
| Total | 22,879 (100.0) | 12,000 (100.0) | 2,074 (100.0) | 1,577 (100.0) |
| Sex |  |  |  |  |
| Female | 10,942 (47.8) | 6,349 (52.9) | 669 (32.3) | 544 (34.5) |
| Male | 11,937 (52.2) | 5,651 (47.1) | 1,405 (67.7) | 1,033 (65.5) |
| Age |  |  |  |  |
| 18 - 34 | 10,516 (46.0) | 5,222 (43.5) | 638 (30.8) | 496 (31.5) |
| 35 - 49 | 6,823 (29.8) | 3,833 (31.9) | 766 (36.9) | 605 (38.4) |
| 50 - 64 | 5,540 (24.2) | 2,945 (24.5) | 670 (32.3) | 476 (30.2) |
| Race/Ethnicity |  |  |  |  |
| White, Non-Hispanic | 7,718 (33.7) | 4,370 (36.4) | 871 (42.0) | 672 (42.6) |
| Hispanic/Latinx | 9,106 (39.8) | 4,824 (40.2) | 605 (29.2) | 489 (31.0) |
| Black/African American, Non-Hispanic | 4,140 (18.1) | 2,043 (17.0) | 494 (23.8) | 342 (21.7) |
| Other Non-Hispanic/Unknown | 1,915 (8.4) | 763 (6.4) | 104 (5.0) | 74 (4.7) |
| Language |  |  |  |  |
| English | 20,674 (90.4) | 10,971 (91.4) | 2,052 (98.9) | 1,559 (98.9) |
| Spanish | 1,774 (7.8) | 812 (6.8) | 19 (0.9) | 15 (1.0) |
| Other/Unknown | 431 (1.9) | 217 (1.8) | 3 (0.1) | 3 (0.2) |
