# Supplementary material for: Minimizing H.R.1-related Medicaid coverage disruptions for high-risk patients
Source: Health Aff Sch. 2026 Jul 1;4(7):qxag162. doi: 10.1093/haschl/qxag162 (PMC13367571; doi:10.1093/haschl/qxag162)

Figure S1. CONSORT-like documenting how Denver Health 2021 Medicaid attributed enrollees were identified for a case study related to minimizing H.R.1-related coverage disruptions for high-risk populations.


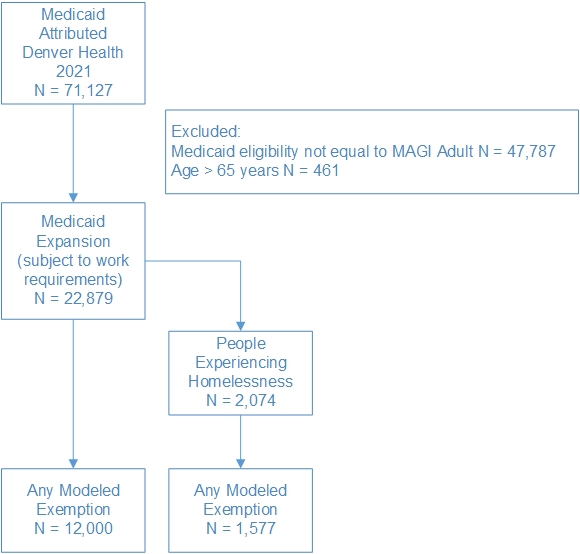

Supplement: qxag162_Supplementary_Data [file qxag162_supplementary_data.zip › Figure_S1.docx]
